# Associations of adolescent menstrual symptoms with school absences and educational attainment: analysis of a prospective cohort study

**DOI:** 10.1038/s41539-025-00338-x

**Published:** 2025-08-19

**Authors:** Gemma Sawyer, Abigail Fraser, Deborah A. Lawlor, Gemma C. Sharp, Laura D. Howe

**Affiliations:** 1 MRC Integrative Epidemiology Unit, University of Bristol, Bristol, England UK; 2 Population Health Sciences, Bristol Medical School, University of Bristol, Bristol, England UK; 3 School of Psychology, Faculty of Health and Life Sciences, University of Exeter, Exeter, England UK

**Keywords:** Education, Human behaviour

## Abstract

Menstrual symptoms may negatively impact academic achievement, but rigorous population-based studies are lacking. 2,698 participants from the Avon Longitudinal Study of Parents and Children (ALSPAC) self-reported heavy or prolonged bleeding and menstrual pain during adolescence and multivariable regression were used to estimate associations with linked data on absences and attainment at age 15/16, adjusting for confounders. Heavy or prolonged bleeding and pain were associated with missing 1.7 (16.58% increase) and 1.2 (12.83% increase) additional days of school per year, respectively, and 48% and 42% higher odds of persistent (≥10%) absence. Heavy or prolonged bleeding was associated with lower examination scores (−5.7 points) and 27% lower odds of achieving five standard passes. The association between pain and attainment was weaker but still present (−3.14 points; 95% CI: −7.46, 1.17; 16% lower odds of five standard passes). Greater research and support are needed to prevent adolescents’ menstrual symptoms impacting their academic achievement.

## Introduction

Menstrual symptoms, such as pain (dysmenorrhea) and heavy menstrual bleeding (HMB) affect a high proportion of girls and people who menstruate and may negatively impact their wellbeing, ability to attend school, and fulfil their academic potential^1–4^. A systematic review of cross-sectional studies reported that 12% of school/university students in high-income countries, and 26% in low- and middle-income countries, have been absent from school due to dysmenorrhea and up to 41% of students reported that menstrual pain negatively impacts their performance^5^. Other reviews of dysmenorrhea have supported the notion that menstrual pain is a leading reason for absenteeism, with between 10 and 30% missing up to two days each month^1,3^. Dose-response relationships, whereby higher pain scores are associated with more time off school or worsened performance, have also been demonstrated through a cross-sectional survey in Australia^6^. A large cross-sectional survey in women of reproductive age (15–45 years) from the Netherlands calculated that menstruation-related symptoms were associated with an average of 1.3 days lost every year due to absences and 8.9 days lost each year due to impacted productivity^7^. A small number of surveys in Japan and Sweden have also explored HMB and demonstrated associations with more absences from work and impaired productivity^8,9^. Thus, absences and impaired ability to engage with schoolwork because of difficulties concentrating, fatigue, and fear of leaking could lead to effects of menstrual symptoms on educational attainment^10,11^.

However, there are flaws with the existing literature on menstrual symptoms and school attendance and attainment. Most research focuses on pain or menstruation-related symptoms broadly, meaning understanding regarding other menstrual symptoms has been restricted. HMB is one important symptom where the literature is comparatively scarce despite contribution to concerns around leaking or failures to conceal menstruation, a greater need for access to toilets and menstrual products, and physical and cognitive symptoms related to iron deficiency^2^. The focus on menstrual-related symptoms or difficulties more broadly has also limited understanding because different symptoms may be associated in differing ways and have different pathways through which they are associated with attendance and/or productivity. The research also primarily relies on self-reported absences where students may underreport because they either do not feel comfortable disclosing the reason for their absence or may not be aware that their menstruation has resulted in them feeling unwell (e.g., fatigue from iron deficiency anaemia because of HMB)^2^. It would therefore be useful to examine how menstrual symptoms relate to school-recorded, as opposed to self-reported, absences. Similarly, previous research has focused on self-reported concentration or productivity^5^. Whilst it is important to research perceived experiences, it would also be useful to examine the relationship between menstrual symptoms and examination results to understand how experiences of reduced productivity translate into educational attainment.

The majority of previous research consists of cross-sectional surveys recruited specifically with a focus on menstruation where participants simultaneously self-reported their menstrual symptoms and absences/productivity, increasing the likelihood of selection bias and overreporting of menstrual-related impacts^5^. Examining this relationship longitudinally, within a population sample, where menstrual symptoms have been reported prior to absences/productivity will minimise these issues. Finally, as few studies have adjusted for key confounders, notably socioeconomic position and early life mental health, it is unclear whether relationships between menstrual symptoms and educational outcomes are spurious.

The current study aims to explore the associations between HMB and menstrual pain, and school absences and educational attainment whilst addressing limitations of existing evidence.

## Results

Of 2698 participants, 972 (36%) reported heavy or prolonged bleeding and 1496 (55%) reported menstrual pain. Across the sample, the mean GCSE points score was 352.89 (SD 73.30) and the median percentage absent was 5.37% (IQR 2.61, 9.46). 68.9% of participants achieved five A*-C GCSE grades and 23.1% were ‘persistently absent’ (≥10%) (Table 1). Supplementary Table 1 shows the proportion or mean of outcomes and confounders for the groups with and without each menstrual symptom. The participants included in the analyses tended to be of higher socioeconomic position than those excluded due to missing data (Supplementary Table 2).

**Table 1:** Descriptive statistics for exposure, outcomes, and covariates, alongside the percentage of missing data before multiple imputation in the full sample (*N* = 2698)

Variable	Prevalence	Percentage missing prior to MI
Exposures		
Heavy or prolonged bleeding	36.0%	0
Menstrual-related pain	55.5%	0
Continuous outcomes		
Median percentage absence (IQR)	5.37 (2.61, 9.46)	0
Mean GCSE score (SD)	352.89 (73.30)	0
Binary outcomes		
Persistent absence (≥10%)	23.1%	0
Five A*-C GCSEs including Maths and English	68.9%	0
Sociodemographic factors		
Non-white ethnicity	3.8%	9.2
Maternal education		
CSE/Vocational	22.3%	12.0
O level	38.1%	
A level	25.6%	
Degree	14.0%	
Manual parental social class	47.7%	12.2
Any financial difficulty	61.0%	10.3
Renter or non-homeowner	13.8%	8.1
Maternal smoking pre-pregnancy	27.1%	6.8
Adverse childhood experiences		
Parental separation before age 11	23.4%	12.1
Physical abuse before age 11	38.4%	13.0
Sexual abuse before age 11	3.5%	13.8
Maternal depression at 12.1 years	23.6%	21.9
Child factors		
Mean age at menarche in years (SE)	12.66 (0.024)	2.9
Mean BMI (kg/m2) (SE)	20.39 (0.076)	21.4
Mean internalising SDQ score at 9.6 years (SE)	2.63 (0.057)	17.1
Mean externalising SDQ score at 9.6 years (SE)	3.74 (0.060)	17.1
Mean IQ at 8 years (SE)	103.49 (0.331)	22.5
Past year oral contraception at exposure timepoint^a^	14.9%	1.3^a^

*MI* multiple imputation, *GCSE* general certificate of secondary education, *SE* standard error, *IQR* interquartile range, *CSE* certificate of secondary education, *BMI* body mass index, *SDQ* strengths and difficulties questionnaire, *IQ* intelligence quotient.

^a^Contraception was not imputed.

### Menstrual symptoms and school absences

**Fig. 1:**
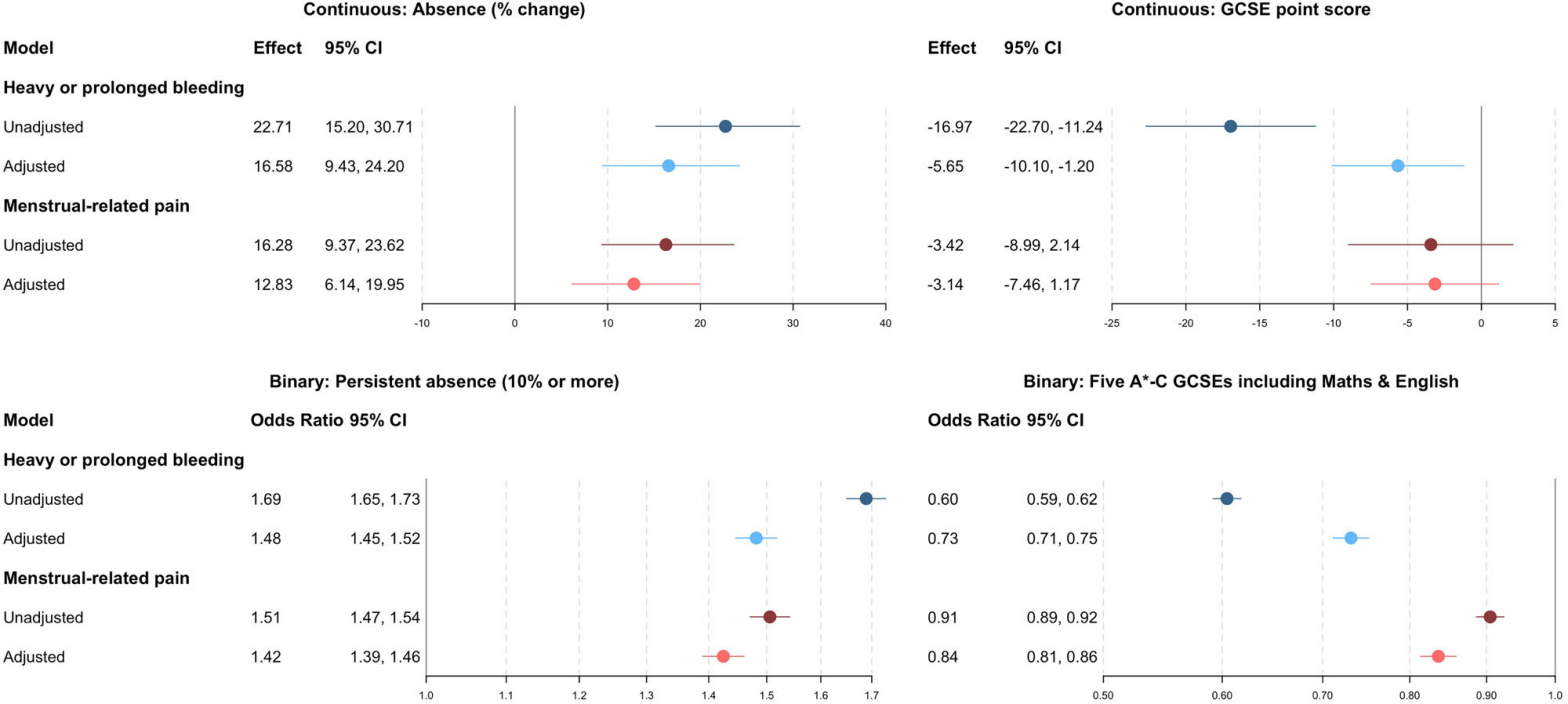
Linear regression analysis of the association between menstrual symptoms and GCSE score and school absences and logistic regression analysis of the association between menstrual symptoms and achieving five A*-C GCSEs including Maths and English and persistent absence (10% or more) (*N* = 2698). GCSE general certificate of secondary education, CI confidence interval. Adjusted for ethnicity; maternal education, parental social class, financial difficulties, and home ownership during pregnancy; maternal smoking pre-pregnancy; parental separation, physical abuse, and sexual abuse before 11; maternal depression at 12.1; age at menarche; body mass index at 12.8; internalising and externalising problems at 9.6; and intelligence quotient at 8.

Participants reporting heavy or prolonged bleeding had, on average, 16.6% (95% CI: 9.4, 24.2) more time off school (equating to 1.7 days more) compared to those who had never experienced heavy or prolonged bleeding, after adjusting for all confounders. Participants reporting severe menstrual pain had, on average, 12.8% (95% CI: 6.1, 20.0) more time absent from school (1.2 days more). When exploring persistent absence (i.e., 10% or more), heavy or prolonged bleeding (OR 1.48; 95% CI: 1.45, 1.52: absolute risk difference 9.6%) and menstrual pain (OR 1.42; 95% CI: 1.39, 1.46: absolute risk difference 7.2%) were associated with an increased likelihood of being persistently absent (Fig. 1). The results from the unadjusted analyses supported the same conclusions.

### Menstrual symptoms and educational attainment

Participants reporting heavy or prolonged bleeding had a lower total GCSE score (−5.65; 95% CI: −10.10, −1.20) compared with those who did not report heavy or prolonged bleeding, after adjusting for confounders (equivalent to roughly a 1 grade reduction, i.e., moving from an A to a B). There was less evidence that menstrual pain (−3.14; 95% CI: −7.46, 1.17) was associated with a difference in GCSE score. There was evidence that heavy or prolonged bleeding (OR 0.73; 95% CI: 0.71, 0.75: absolute risk difference −5.9%) and menstrual pain (OR 0.84; 95 CI: 0.81, 0.86: absolute risk difference −2.2%) were associated with a reduced likelihood of achieving five A*-C GCSEs including Maths and English (Fig. 1). The results from the unadjusted analyses support the same conclusions.

### Complete case

The full imputed and complete case results are presented in Supplementary Tables 3–7. The complete case results were consistent with the imputed results for the continuous outcomes and the association between menstrual pain and persistent absence. The associations between heavy or prolonged bleeding and binary outcomes were attenuated when adjusting for confounders in the complete case analysis, and there was no evidence for an association between menstrual pain and achieving five A*-C grades.

### Oral contraceptive use

When stratifying by past-year oral contraceptive use, menstrual pain was associated with higher rates of absence in participants not using contraception (*N* = 1093; 12.0%; 95% CI: 2.5, 22.5), but not in those using contraception (*N* = 165; −2.0%; 95% CI: −24.3, 26.9), although the CIs overlapped and there was no statistical evidence of interaction. For all other exposure-outcome pairs, results were similar in participants who were or were not using oral contraception (Table 2).

**Table 2:** Linear regression analysis of the associations between menstrual symptoms and school absences and GCSE score stratified by past-year oral contraceptive use in the sample providing data on oral contraceptive use (*N* = 2664)

	School absence in Year 11				GCSE points score			
	% change	95% CI	*P* value	Interaction *p* value	Beta	95% CI	*P* value	Interaction *p* value
Heavy or prolonged bleeding								
Crude analysis (*N* = 2664)								
Data on oral contraceptive use (*N* = 2664)	22.41	14.87, 30.44	<0.001	0.882	−17.36	−23.12, −11.60	<0.001	0.756
No oral contraceptive use (*N* = 2257)	16.11	8.26, 24.52	<0.001		−14.59	−20.83, −8.35	<0.001	
Oral contraceptive use (*N* = 407)	17.63	0.03, 38.33	0.050		−12.11	−28.44, 4.22	0.146	
Adjusted analysis (*N* = 1258)								
Data on oral contraceptive use (*N* = 1258)	11.27	1.74, 21.70	0.020	0.710	−6.31	−11.83, −0.79	0.025	0.660
No oral contraceptive use (*N* = 1093)	7.79	−2.22, 18.84	0.131		−5.21	−11.07, 0.65	0.081	
Oral contraceptive use (*N* = 165)	14.92	−8.93, 45.02	0.239		−2.55	−19.00, 13.90	0.760	
Menstrual pain								
Crude analysis (*N* = 2664)								
Data on oral contraceptive use (*N* = 2664)	16.33	9.38, 23.71	<0.001	0.551	−3.10	−8.69, 2.50	0.278	0.848
No oral contraceptive use (*N* = 2257)	12.24	5.14, 19.82	0.001		−0.24	−6.09, 5.61	0.935	
Oral contraceptive use (*N* = 407)	13.44	−4.93, 35.37	0.161		−3.75	−21.55, 14.06	0.680	
Adjusted analysis (*N* = 1258)								
Data on oral contraceptive use (*N* = 1258)	11.47	2.41, 21.34	0.012	0.251	−2.61	−7.84, 2.62	0.328	0.296
No oral contraceptive use (*N* = 1093)	12.03	2.45, 22.50	0.013		−2.35	−7.74, 3.03	0.392	
Oral contraceptive use (*N* = 165)	−1.97	−24.29, 26.91	0.879		2.59	−15.58, 20.77	0.778	

In participants with complete data on oral contraceptive use, we stratified the analyses by past year oral contraceptive use (‘oral contraceptive use’ or ‘no oral contraceptive use’) and used a likelihood ratio test to assess statistical evidence of interaction. Abbreviations: GCSE, general certificate of secondary education; CI, confidence interval. Adjusted for ethnicity; maternal education, parental social class, financial difficulties, and home ownership during pregnancy; maternal smoking pre-pregnancy; parental separation, physical abuse, and sexual abuse before 11; maternal depression at 12.1; age at menarche; body mass index at 12.8; internalising and externalising problems at 9.6; and intelligence quotient at 8.

### Additional analyses

The results were consistent with the main results and support similar conclusions. Details can be found in Supplementary Material (pp 4–6).

## Discussion

This study provides evidence that heavy or prolonged bleeding and menstrual pain in adolescence are associated with greater time absent from school and lower educational attainment.

Our findings are consistent with previous research reporting relationships between HMB and school/work performance^8^, and between menstrual pain, school absenteeism^5,12^ and productivity^5,7^, supporting the consistency and strength of such relationships. The results suggest that menstrual symptoms are associated with 1–2 more days absent during the final year of schooling and, whilst meaningful, it is unlikely this fully explains the association with attainment, supporting previous literature that menstrual symptoms can impact concentration at school^7^. While we found evidence of an association between menstrual pain and attaining five or more good GCSEs, we did not find strong evidence for an association with GCSE point score (as a continuum). The reasons for this discrepancy are unclear. There is some variation in the prevalence of menstrual symptoms; 55% reported menstrual pain in this study compared with 71% in a previous systematic review^5^ and 36% reported heavy or prolonged bleeding compared with 19%^8^. These differences may reflect different measurement approaches, contexts, or recall periods; however, the current prevalence falls within ranges identified by wider menstrual health literature^1,13^.

This study’s strengths include the population-based prospective cohort design, use of objective outcome measures, and comprehensive adjustment for confounders. Moreover, heavy or prolonged bleeding was self-reported, which is increasingly recognised as the most appropriate measure and used in the majority of research in this field^14,15^. However, whilst we attempted to minimise loss to follow up and missing data by using linked education data and multiple imputation of confounders to maintain a larger and more representative sample, only 37% of the full female sample was included. Moreover, participants were asked whether they experienced ‘heavy or prolonged bleeding’; this limits our ability to determine which of these two, separate clinical symptoms drive the observed associations^15^. When attempting to incorporate additional information on number of days bleeding in an additional analyses, the results suggests that heavy but not prolonged bleeding was associated with more time absent. Results were less clear for educational attainment, potentially due to lower statistical power, and ‘prolonged bleeding’ was defined as 7 days or more in this analysis, because this was the highest response category, even though prolonged bleeding is defined as 9 days or more clinically^15^.

The menstrual symptom definitions in this study were also somewhat limited as participants were asked whether they had *ever* been experienced, meaning participants may no longer be experiencing symptoms at the time of outcome assessment and we were unable to distinguish those with persistent, occasional, or resolved symptoms. The definitions were also binary, meaning the groups are heterogenous in terms of symptom severity and underlying cause. Some participants’ symptoms may have been due to underlying gynaecological disorders, such as endometriosis, polycystic ovary syndrome, or fibroids, which may impact educational outcomes through pathways other than measured symptoms (for example, irregular cycles, fatigue, and gastrointestinal symptoms)^16^. Unfortunately, data was not available to assess these disorders, although it is likely few would have been diagnosed by age 16 due to underdiagnosis and long delays between symptom onset and diagnosis^17^. We attempted to address this in additional analyses, which generally showed educational consequences were more severe for people who consulted a doctor about their symptoms. However, health seeking behaviour may be reflective of other factors such as cultural attitudes, menstrual health knowledge, and socioeconomic position^18,19^. Finally, we explored whether the results differed by contraception use because contraception may be prescribed to alleviate symptoms and we therefore hypothesised that educational impacts of menstrual symptoms may be weaker in people using contraception. We found no statistical evidence of interaction, although these analyses lacked statistical power, making it challenging to draw conclusions and warranting further exploration in larger samples. Finally, although the binary outcomes were relatively common, we adopted to report ORs instead of risk ratios (RRs) due to their mathematical properties (see full explanation in the Supplementary Material pages 12–13) and therefore it is important that the estimates are not incorrectly interpreted as RRs.

Whilst these findings are based upon participants who sat their GCSEs between 2006 and 2009, surveys of UK students conducted between 2019 and 2023 suggest that menstrual difficulties continue to negatively impact attendance and performance^12,20,21^. This highlights the need for further evidence to identify mechanisms underlying the observed associations, possibly including menstrual anxiety and concerns about leaking, bullying, challenges managing symptoms in school, and impacts of iron deficiency anaemia^10,12,21,22^. Research has demonstrated broader negative menstrual experiences, such as lacking information and support, period product insecurity, and gender-based stigma, which may also contribute^11^. We discussed our findings with a patient and public involvement group of adolescent girls currently in secondary school, who reported difficulties focusing during menstruation, restricted toilet access due to school behavioural policies, embarrassment and stigma, and insufficient period product provision. Exploring such potential mechanisms will aid in the development of school-, family-, and community-based interventions.

In summary, our study suggests that heavy or prolonged bleeding and menstrual pain, are associated with lower school attendance and educational attainment. More research is needed to understand the mechanisms behind these associations, and to develop public health measures to mitigate negative impacts of menstrual symptoms on education and tackle menstruation-related inequalities.

## Methods

### Study design

The Avon Longitudinal Study of Parents and Children (ALSPAC) is a longitudinal birth cohort that recruited pregnant women resident in Avon, UK with expected delivery dates between April 1991 and December 1992. The initial number of pregnancies enrolled was 14,541, with 13,988 children alive at age 1. When the oldest children were ~7 years of age, an attempt was made to bolster the initial sample with eligible cases who had not joined originally. Including such children, the total cohort size is 14,901 children who were alive at 1 year of age.

At age 18, participants were sent ‘fair processing’ materials describing ALSPAC’s intended use of their administrative records and were given clear means to consent or object via a written form. Administrative data were not extracted for participants who objected, or who were not sent fair processing materials. Further details on ALSPAC have been published elsewhere^23,24^. The study website contains details of all data through a fully searchable data dictionary and variable search tool: http://www.bristol.ac.uk/alspac/researchers/our-data/. Ethical approval for the study was obtained from the ALSPAC Ethics and Law Committee and the Local Research Ethics Committees (NHS Haydock REC: 10/H1010/70). Informed consent for the use of data collected via questionnaires and clinics was obtained from participants following the recommendations of the ALSPAC Ethics and Law Committee at the time.

### Participants

**Fig. 2:**
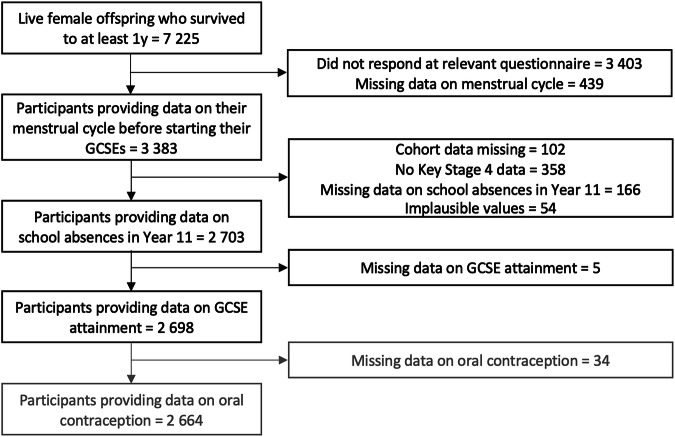
Flow diagram of the Avon longitudinal study of parents and children participants into the current study sample. Black boxes represent the sample included in the main analysis and grey boxes show the additional exclusions to establish the sample used in the additional analysis regarding oral contraceptive use.

Participants who were assigned female at birth (*N* = 7225) and had information on exposures and outcomes were eligible for inclusion in this study (*N* = 2698; 37%) (Fig. 2).

### Exposures

Participants were asked about menstruation in nine questionnaires throughout adolescence (8–17 years)^25^. We defined menstrual symptoms based on the measure closest in time, but no more than two years, before January of the final compulsory year of schooling (September–July, age 15/16). Therefore, menstrual data was extracted from one of four questionnaires and participants were aged 13 (*N* = 169), 14 (*N* = 501), 15 (*N* = 996), or 16 (*N* = 1032).

#### Heavy or prolonged bleeding

In the questionnaires, participants were asked “Have you ever had any of the following symptoms associated with your period: heavy or prolonged bleeding?” and could answer either yes or no. We derived a binary heavy or prolonged bleeding variable (‘yes’ or ‘no’).

#### Menstrual-related pain

Depending on which of the four questionnaires was used, participants were either asked “Have you ever had any of the following symptoms associated with your period: severe cramps?” (with response options yes or no) or “Have you ever had any of the following symptoms associated with your period: pain with your period” and “if yes, were they mild, moderate, or severe?”. We derived a binary menstrual pain variable; ‘severe cramps or moderate/severe pain’ or ‘no severe cramps or mild/no pain’^25^.

### Outcomes

Outcome data came from linkage to the National Pupil Database (NPD) (https://find-npd-data.education.gov.uk/). For each outcome, we utilised a continuous (statistical power and granularity) and a binary (recognised metrics to enhance interpretability) definition.

#### Absences

Absences were defined as the number of half day sessions missed (authorised, e.g. illness reported by parents, or unauthorised, e.g. term-time holidays or truancy) divided by the total number of available sessions in the final year of compulsory schooling when pupils take final exit examinations (age 15/16). Participants with implausible values for the number of available sessions (zero or more than 330, *N* = 54) were excluded. We also dichotomised absences based on whether participants were persistently absent (10% or more), following the definition monitored at a school level in the UK^26^.

#### Educational attainment

Educational attainment was based on GCSE (General Certificate of Secondary Education) qualifications, which are compulsory qualifications in a range of subjects usually taken at age 15/16. At the time GCSEs were completed by this cohort, they were graded A* (highest) to G (grade C reflects a standard pass), and U (unclassified). We used GCSE and equivalent total point score, which is a continuous measure (range 0–540), using up to eight highest GCSE grades, where six points are given for each grade increase (grades and score: A* = 58; A = 52; B = 46; C = 40; D = 34; E = 28; F = 22; G = 16; U = 0). Scores above 464 (eight A* grades) reflect pupils who took Advanced Subsidiary level (AS-level) exams early (advanced qualifications usually taken the year after GCSEs for those who continue in education). We also used a binary educational attainment outcome reflecting whether participants achieved five A*-C GCSEs (standard passes), including Maths and English, which was an important performance indicator and a frequently used criteria for further education^27^.

### Confounders

We selected confounders that could plausibly cause both menstrual symptom exposures (or self-reported values) and educational outcomes, including ethnicity^28^, socioeconomic position^28,29^, childhood adversity^30,31^, child and maternal mental health^29,32^, body mass index (BMI)^1,29,33^, intelligence quotient (IQ)^34,35^, and age at menarche^1,29,33,36^. This is particularly challenging to determine for menstrual symptom exposures as the literature is somewhat limited; however, we have opted to be inclusive in our confounder selection as including a variable that is only related to the outcome (and not on the causal pathway between exposure and outcome, i.e., a competing exposure) would increase precision and not bias the association^37^. Therefore, whilst we conceptualise all variables included as confounders; we recognise that they could be competing exposures (see Supplementary Fig. 1).

We included multiple socioeconomic factors reported by mothers via questionnaires during pregnancy, including occupation (dichotomised into ‘manual’ or ‘non-manual’ based on the 1991 British Office of Population and Census Statistics classification), maternal education (‘Certificate of Secondary Education/vocational’, ‘O level’, ‘A level’, or ‘degree’), home ownership (‘owner or private renter’ or ‘renter or non-homeowner’), financial difficulties score (dichotomised to ‘any difficulties (score of 1 or above)’ or ‘no difficulties (score of 0)), and smoking pre-pregnancy (‘any smoking’ or ‘no smoking’) as confounders. Other confounders include child ethnicity (reported by mothers via questionnaire during pregnancy; ‘white’ or ‘non-white’), age at menarche (mother- or self-reported via questionnaires during puberty), body mass index (BMI (kg/m^2^); measured at a clinic visit) at 12.8 years, maternal-reported depression in the past 2 years (‘yes’ or ‘no’) collected via questionnaire when the participant was aged 12.1 years, internalising and externalising problems at 9.6 years (each with a score ranging from 0 to 20 and measured with the Strengths and Difficulties Questionnaire; measured at clinic visit)^38^, and intelligence quotient at age 8 (measured with the Weschler Intelligence Scale for Children at clinic visit)^39^. Adverse childhood experiences (ACEs), including parental separation, sexual abuse, and physical abuse before age 11, were either reported prospectively by their mother or retrospectively self-reported in adulthood. Multiple questionnaire items corresponded to each ACE (see Supplementary Table 8 for details). Binary variables for each ACE construct (‘any’ or ‘none’) were derived for participants who responded to at least 50% of the relevant questions for a given ACE; for participants with fewer than 50% of the relevant questions the ACE was coded as missing^40^.

### Statistical analysis

Analyses were conducted in Stata (version 18.0)^41^. We present descriptive statistics (prevalence or mean) for outcomes and confounders, separately for those who did and did not experience each menstrual symptom. Multivariable linear regression models were used to estimate the association between each menstrual symptom and the continuous outcomes: school absence and GCSE score, adjusting for all confounders. We report the coefficient, 95% confidence intervals (CIs), and *p* values. Absences were defined as the percentage of sessions absent. This variable had a positively skewed distribution, so we performed a log-transformation, meaning the regression coefficients represent the ratio of geometric means of absences comparing those with and without the menstrual symptom. We exponentiate these ratios of geometric means, and the resultant coefficient can be interpreted as the percentage difference in school absences between the exposed and unexposed groups. When GCSE score is the dependent variable, the regression coefficients represent the difference in score between the exposed and unexposed groups. For the binary outcomes (‘persistent absence’ and ‘achieved 5A*-C GCSEs including English and Maths’), multivariable logistic regression models were conducted to estimate the association between the exposures and outcomes, adjusting for all confounders. We report the odds ratios (ORs), 95% CIs, and *p* values. Logistic regression was selected due to the mathematical properties of ORs compared with risk ratios (RRs; outlined in the Discussion); however, estimates cannot be interpreted as RRs as the outcomes are relatively common^42^.

### Missing data

We did not impute exposures and outcomes due to uncertainty about the ability to predict missing values of menstrual symptoms, and high data availability in outcomes due to the use of linked data. Of 2698 participants included in our main analyses (with complete data on all exposures and outcomes; Figs. 1), 1424 (52.8%) had missing data on at least one confounder. We used multiple imputation (MI) to address missing confounder data (Table 1). We included all observed variables used in analyses, including exposures and outcome, in MI equations. Additional auxiliary variables, including maternal depression during pregnancy (measured with the Edinburgh Postnatal Depression Scale)^43^, BMI at age 7, and SDQ at age 11, were used to impute maternal depression, BMI, and internalising and externalising problems, respectively. Results were calculated across 60 impute datasets, guided by results of Monte Carlo error tests (which assess statistical reproducibility of the imputation^44^) and associations were generated by pooling across these datasets using Rubin’s rules^45^. The prevalence/means of each confounder before and after MI are presented in Supplementary Table 2.

### Oral contraception

Oral contraceptives are often prescribed to ameliorate menstrual symptoms^46,47^ and could potentially impact absences or attainment indirectly through mood, physical, or cognitive side effects. It is challenging to identify if and how contraception should be accounted for in this analysis as we do not have data on the relative timing of contraceptive use and menstrual symptoms; however, it is more likely the menstrual symptom preceded contraceptive use as participants are reporting whether they have *ever* experienced menstrual symptoms (compared with contraception use in the last 12 months), meaning adjusting for contraceptive use would be inappropriate over-adjustment (adjusting for a variable on the causal pathway between exposure and outcome). However, contraceptive use may modify the effect of menstrual symptoms on educational outcomes, i.e., in people who report *ever* experiencing the symptom but whose symptoms have improved following contraception initiation, educational consequences may be diminished. Therefore, in a sensitivity analysis, we stratified the analyses by past year oral contraceptive use (‘use’ or ‘no use’), which was self-reported in the same questionnaire as the participants reported their menstrual symptoms. This was conducted in participants with complete data on contraceptive use only (Fig. 1), using a likelihood ratio test to assess statistical evidence of interaction.

### Additional analyses

We conducted a series of additional analyses to explore whether our results were influenced by the specific definitions of menstrual symptoms, minimise residual confounding, and exclude unauthorised absences.

#### Alternative menstrual symptom exposure definitions

Using the imputed data, we conducted confounder adjusted linear regression to assess the associations between four exposures and the continuous outcomes (percentage absence and GCSE points score):

- A three-level variable based on whether participants went to the doctor for heavy or prolonged bleeding (‘heavy or prolonged bleeding and went to the doctor’, ‘heavy or prolonged bleeding but did not go to the doctor’, or ‘no heavy or prolonged bleeding’) to explore whether any effects of heavy or prolonged bleeding are more severe for participants who sought medical help.
- A three-level variable based on whether participants went to the doctor for menstrual pain (‘pain and went to the doctor’, ‘pain but did not go to the doctor’, or ‘no pain’) to explore whether any effects of menstrual pain are more severe for participants who sought medical help.
- A four-level variable to separate heavy bleeding from prolonged bleeding. Participants were asked how many days bleeding they usually have during each period and were able to report the exact number of days or, if they were unsure, select one of three categories: 3 days or less, 4–6 days, or 7 days or more. Prolonged bleeding was defined as ‘7 days or more’ if participants reported bleeding for 7 days or more in either the categorial or continuous response option and ‘less than 7 days’ if participants reported 6 days bleeding or less in either response option (no participants responded to both the categorical and continuous option). This was used alongside responses to the main heavy or prolonged bleeding variable to derive a four-level variable: ‘heavy and prolonged bleeding’ (‘yes’ to heavy or prolonged bleeding AND reporting 7 days or more bleeding), ‘heavy bleeding only’ (‘yes’ to heavy or prolonged bleeding, BUT reporting 6 or fewer days bleeding), ‘prolonged bleeding only’ (‘no’ to heavy or prolonged bleeding, BUT reporting 7 days or more bleeding), or ‘neither heavy nor prolonged’ (‘no’ to heavy or prolonged bleeding AND reporting 6 days or fewer bleeding’).
- A four-level variable to explore the effects of co-occurring menstrual symptoms: ‘heavy bleeding and pain’, ‘heavy bleeding only’, ‘pain only’, and ‘neither heavy bleeding nor pain’.

#### Prior educational attainment

To minimise residual confounding, we adjusted for attainment in Key Stage 1 (KS1) Standard Assessment Tests (SATs). These were completed at age 6/7 years, which is prior to menarche for all participants. We used SATs summary score, which is a continuous measure (range 0–15) where higher scores reflect higher levels of attainment, derived by summing scores obtained in reading, writing, and mathematics (each ranging from 0 to 5). Further detail can be found on the National Pupil Database (NPD) (https://find-npd-data.education.gov.uk/).) In the complete case sample with KS1 data available (*N* = 1120), we conducted the main multivariable linear and logistic regression models adjusting for all confounders with and without additional adjustment for KS1 attainment.

#### Authorised absences

The definition of absences used in our main continuous and binary analyses include both authorised and unauthorised (including term-time holidays and truancy) absences; however, it is possible that these could be associated differently with menstrual symptoms. As a sensitivity analysis, therefore, we conducted the multivariable linear and logistic regression models for absences, including only authorised absences in the main definition (excluding truancy and term-time holiday) in the complete case sample (*N* = 1274).

We have followed the STROBE (Strengthening the Reporting of Observational Studies in Epidemiology) guidelines in the reporting of this study (supplement pp 13–16)^48^.

## Supplementary information

Supplementary Material

## Data Availability

ALSPAC data access is through a system of managed open access. The steps below highlight how to apply for access to the data included in this paper and all other ALSPAC data. 1. Please read the ALSPAC access policy (http://www.bristol.ac.uk/media-library/sites/alspac/documents/researchers/data-access/ALSPAC_Access_Policy.pdf) which describes the process of accessing the data and samples in detail, and outlines the costs associated with doing so. 2. You may also find it useful to browse the fully searchable research proposals database (https://proposals.epi.bristol.ac.uk/), which lists all research projects that have been approved since April 2011. 3. Please submit your research proposal (https://proposals.epi.bristol.ac.uk/) for consideration by the ALSPAC Executive Committee. You will receive a response within 10 working days to advise you whether your proposal has been approved. 4. If you have any questions about accessing data or samples, please email alspac-data@bristol.ac.uk (data) or bbl-info@bristol.ac.uk (samples).

## Code availability

Analysis code is available on GitHub at https://github.com/GemmaS17 (Stata, version 18.0).
