# Supplementary material for: Associations of adolescent menstrual symptoms with school absences and educational attainment: analysis of a prospective cohort study
Source: NPJ Sci Learn. 2025 Aug 19;10:54. doi: 10.1038/s41539-025-00338-x (PMC12365126; doi:10.1038/s41539-025-00338-x)
Supplement: Supplementary file 1 — Supplementary Material [file 41539_2025_338_MOESM1_ESM.pdf]

<u>Supplementary Material</u>

Results

Additional analyses in the imputed data

a) Seeking medical care for heavy or prolonged bleeding

The association between heavy and prolonged bleeding and school absences was stronger for those who had seen a doctor about their symptoms (42.6%, 95% CI: 26.4, 60.9; equivalent to 4.1 days) compared with those who reported heavy and prolonged bleeding but had not consulted a doctor (10.9%, 95% CI: 3.7, 18.7; equivalent to 1.0 day). In contrast, the association between heavy and prolonged bleeding with educational attainment was similar whether or not people who had consulted a doctor (-5.5; 95% CI: -10.2, -0.7 versus -5.9; 95% CI: -14.6, 2.7) (Supplementary Figure 2).

b) Seeking medical care for menstrual pain

The association of menstrual pain with absence was stronger in those who had consulted a doctor compared with those who had not consulted a doctor (42.3%; 95% CI: 26.7, 59.8, equivalent to 3.7 days versus 8.8%; 95% CI: 2.1, 15.8, equivalent to 0.8 days). For pain, the effect estimates were slightly larger for those that had compared with had not consulted a doctor (-6.8; 95% CI: -15.0, 1.5 versus -2.5; 95% CI: -7.0, 1.9), although the CIs crossed the null for both groups (Supplementary Figure 2).

c) Separated heavy bleeding and prolonged bleeding

Participants with ‘heavy bleeding only’ (14.7%; 95% CI: 6.8, 23.1, equivalent to 1.5 days) and ‘heavy and prolonged bleeding’ (17.7%; 95% CI: 5.7, 31.1, equivalent to 1.9 days) were absent from school more than participants who had neither symptom. The effect estimate for participants with ‘prolonged bleeding only’ was in the opposite direction, but the CIs were wide and crossed the null (-7.5%; 95% CI: -17.4, 3.5). Similarly, the GCSE score for ‘prolonged bleeding only’ was higher compared with the reference group of neither symptom (5.2; 95% CI: -2.8, 13.5), and lower for ‘heavy bleeding only’ (-4.8; 95% CI: -9.8, 0.2), or ‘heavy and prolonged bleeding’ (-5.6; 95% CI: -13.2, 2.1); however, all CIs were wide and crossed the null (Supplementary Figure 2).

d) Co-occurrence of heavy or prolonged bleeding and pain

The results suggested that, compared with participants with neither heavy nor prolonged bleeding nor pain, participants in the ‘pain only’ group were absent 8.8% (95% CI: 0.9, 17.4, equivalent to 0.8 days) more, the ‘heavy or prolonged bleeding only’ group 14.2% (95% CI: 1.8, 28.1, equivalent to 1.3 days) more, and the ‘heavy or prolonged bleeding and pain’ group 23.1% (95% CI: 14.0, 33.0, equivalent to 2.2 days) more. There was also evidence that participants in the ‘heavy or prolonged bleeding only’(-8.2; 95% CI: -16.3, 0.0) and ‘heavy or prolonged bleeding and pain’ (-6.5; 95% CI: -11.9, -1.1) groups had a lower GCSE score than those with neither symptom. The coefficient for ‘pain only’ (-2.9; 95% CI: -8.2, 2.5) suggested a lower GCSE score, but the CIs crossed the null (Supplementary Figure 2).

Prior educational attainment

Overall, the results were similar in the models that did and did not adjust for KS1 attainment (Supplementary Table 9). The effect estimate for the association between heavy or prolonged bleeding and GCSE points score was slightly smaller when additionally adjusting for KS1 attainment (-5.73 points; 95% CI -11.50, 0.04 versus -3.63 points; 95% CI -9.02, 1.76); however, the confidence intervals crossed and neither model found strong statistical evidence for an association. It is challenging to draw conclusions from these analyses as the statistical power has likely been reduced by the smaller sample size, as demonstrated by the little evidence for an association between heavy or prolonged bleeding and GCSE points score although this was observed in the full complete case sample (N=1274; Supplementary Table 7).

Authorised absences

Compared with the main complete case results (including both authorised and unauthorised), the authorised absence only results were similar for the continuous outcomes, suggesting that heavy or prolonged bleeding and menstrual pain are associated with an increase in the amount of time absent (although the effect estimates for menstrual pain were slightly smaller) (Supplementary Table 10). For the binary outcomes, the ORs were smaller in the authorised absence only analyses compared with the main complete case analyses for heavy or prolonged bleeding (1.19, 95% CI 0.86, 1.65 versus 1.27, 95% CI 0.93, 1.73) and attenuated for menstrual pain (1.27, 95% CI 0.92, 1.74 versus 1.40, 95% CI 1.03, 1.90). These results may imply that some of the association between menstrual symptoms and being persistently absent could have been somewhat driven by a small group with a large number of unauthorised absences.

Supplementary Figure 1. Directed Acyclic Graph (DAG) depicting assumptions about the causal structure between exposures, outcomes, and confounders (or possible competing exposures, indexed with an asterisk).

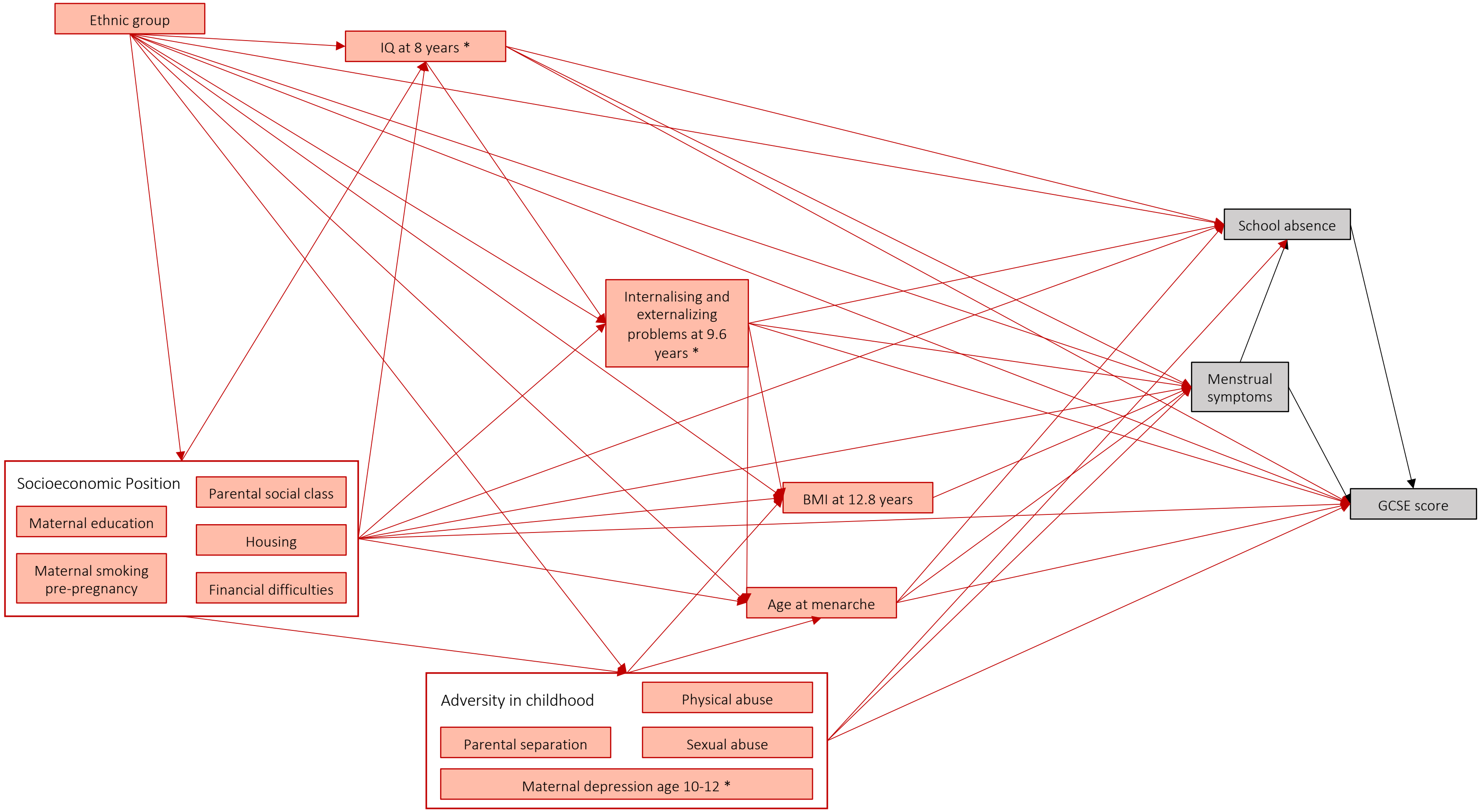

Grey boxes depict the exposures and outcomes (i.e., the association under investigation) and red boxes depict confounders of this relationship. Variables marked with an asterisk are those where there is limited evidence about the presence or nature of association with menstrual symptoms and therefore it is plausible that these could either be confounders (common cause of exposure and outcome) or competing exposures (only cause outcome and not on the causal pathway between exposure and outcome). Adjusting for these variables, regardless of whether they are confounders or competing exposures, is beneficial. Abbreviations: IQ, intelligence quotient; BMI, body mass index; GCSE, general certificate of secondary education.

Supplementary Figure 2. Linear regression analysis of the association between different categories of menstrual symptoms and GCSE score and school absences (N=2698).

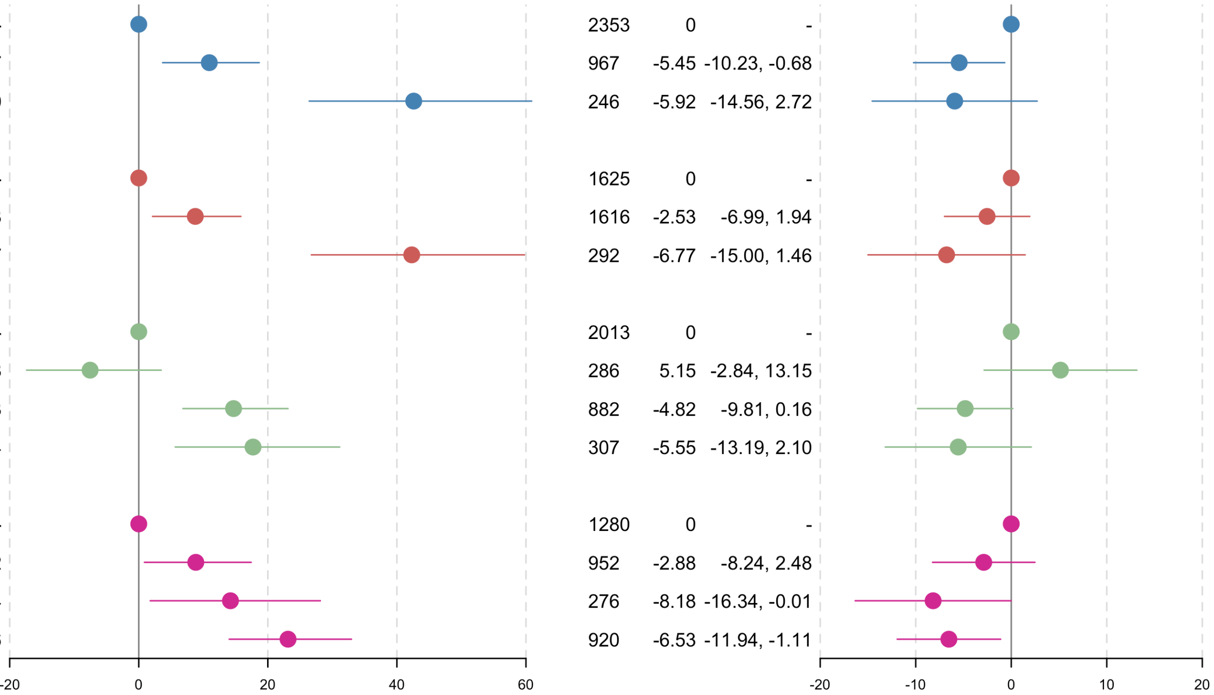

Exposure	Absences (% change) Effect	95% CI	GCSE point score N	Effect	95% CI
**Medical care: heavy or prolonged bleeding**					
No heavy or prolonged bleeding (ref)	0	-	2353	0	-
No medical care	10.93	3.70, 18.67	967	-5.45	-10.23, -0.68
Medical care	42.61	26.41, 60.89	246	-5.92	-14.56, 2.72
**Medical care: menstrual-related pain**					
No menstrual-related pain (ref)	0	-	1625	0	-
No medical care	8.76	2.13, 15.83	1616	-2.53	-6.99, 1.94
Medical care	42.30	26.74, 59.77	292	-6.77	-15.00, 1.46
**Separated heavy and prolonged bleeding**					
Neither (ref)	0	-	2013	0	-
Prolonged bleeding only	-7.54	-17.39, 3.48	286	5.15	-2.84, 13.15
Heavy bleeding only	14.69	6.84, 23.13	882	-4.82	-9.81, 0.16
Both	17.71	5.65, 31.14	307	-5.55	-13.19, 2.10
**Co-occurence of heavy bleeding and pain**					
Neither (ref)	0	-	1280	0	-
Menstrual-related pain only	8.84	0.88, 17.42	952	-2.88	-8.24, 2.48
Heavy or prolonged bleeding only	14.21	1.78, 28.14	276	-8.18	-16.34, -0.01
Both	23.13	14.02, 32.96	920	-6.53	-11.94, -1.11

Abbreviations: GCSE, general certificate of secondary education; CI, confidence interval. Adjusted for ethnicity; maternal education, parental social class, financial difficulties, and home ownership during pregnancy; maternal smoking pre-pregnancy; parental separation, physical abuse, and sexual abuse before 11; maternal depression at 12.1; age at menarche; body mass index at 12.8; internalising and externalising problems at 9.6; and intelligence quotient at 8.

Supplementary Table 1. Distribution of outcomes, sociodemographic factors, adverse childhood experiences, and child factors according to adolescent menstrual symptoms.

	Percentage missing prior to imputation	Heavy or Prolonged Bleeding		Menstrual Pain	
		Yes	No	Yes	No
		972 (36.0%)	1726 (64.0%)	1496 (55.5%)	1202 (44.5%)
**CONTINUOUS OUTCOMES**					
Median percentage absent (IQR)	0	6.33 (3.02, 11.07)	4.85 (2.31, 8.78)	5.74 (2.70, 10.20)	4.89 (2.05, 8.82)
Mean GCSE score (SE)	0	342.03 (2.47)	359.00 (1.70)	351.36 (1.88)	354.00 (2.14)
**BINARY OUTCOMES**					
Persistent absence (≥ 10%)	0	284 (29.2%)	339 (19.6%)	393 (26.3%)	230 (19.1%)
Five A*-C GCSEs including Maths and English	0	601 (61.8%)	1257 (67.7%)	1016 (67.9%)	842 (70.1%)
**SOCIODEMOGRAPHIC FACTORS**					
Non-white ethnicity	9.2	3.7	3.9	3.7	4.0
Maternal education					
CSE/Vocational	12.0	25.4	20.6	22.5	22.1
O level		39.3	37.4	37.2	39.1
A level		25.1	25.8	26.4	24.6
Degree		10.2	16.2	13.9	14.2
Manual parental social class	12.2	51.6	45.5	47.7	47.7
Any financial difficulties	10.3	65.3	58.6	62.0	59.7
Renter or non-homeowner	8.1	16.5	12.3	14.3	13.1
Maternal smoking pre-pregnancy	6.8	31.0	24.9	29.6	24.0
**ADVERSE CHILDHOOD EXPERIENCES**					
Parental separation before age 11	12.1	26.0	22.0	23.5	23.4
Physical abuse before age 11	13.0	37.9	38.6	40.3	36.0
Sexual abuse before age 11	13.8	4.9	2.7	3.4	3.6
Maternal depression at 12.1 years	21.9	30.4	19.7	26.0	20.5
**CHILD FACTORS**					
Mean age at menarche in years *(SE)*	2.9	12.60 (.04)	12.70 (.03)	12.53 (.03)	12.82 (.03)
Mean BMI (kg/m2) at 12.8 years *(SE)*	21.4	20.59 (.14)	20.29 (.09)	20.55 (.10)	20.21 (.12)
Mean internalising SDQ at 9.6 years *(SE)*	17.1	2.78 (.10)	2.54 (.07)	2.80 (.08)	2.41 (.08)
Mean externalising SDQ at 9.6 years *(SE)*	17.1	4.08 (.11)	3.55 (.07)	3.87 (.09)	3.58 (.09)
Mean IQ at 8 years *(SE)*	22.5	102.25 (.56)	104.21 (.41)	103.82 (.44)	103.11 (.51)
Past year oral contraception	1.3*	25.0	9.8	19.6	9.9

*Contraception was not imputed. Abbreviations: GCSE, general certificate of secondary education; SE, standard error; IQR, interquartile range; CSE, certificate of secondary education; BMI, body mass index; SDQ, strengths and difficulties questionnaire; IQ, intelligence quotient.

Supplementary Table 2. Proportion of missing data in the study sample (N=2698); distribution of confounders before and after multiple imputation; and comparison with the sample of female offspring who survived to at least 1 year and were excluded due to missing exposure or outcome data (N=4527).

	Study sample (reporting on exposures and outcomes) N=2698				Excluded due to missing exposures or outcomes N=4527	
	Before multiple imputation			After multiple imputation		
**Variable**	**Proportion / mean (SD)**	**N**	**Proportion missing data**	**Proportion / mean**	**Proportion / mean (SD)**	**N**
Ethnicity	3.72% non-white	2449	9.23%	3.87% non-white	6.01% non-white	3360
Maternal education	21.10% CSE<br>38.16% O level<br>26.16% A level<br>14.57% Degree	2374	12.01%	22.37% CSE<br>38.09% O level<br>25.51% A level<br>14.02% Degree	28.12% CSE<br>35.88% O level<br>22.50% A level<br>13.50% Degree	3222
Parental social class	46.58% manual	2370	12.16%	47.76% manual	51.15% manual	3175
Financial difficulties	60.55% any	2421	10.27%	61.06% any	66.25% any	3396
Housing	13.55% non-homeowner or renter	2480	8.08%	13.79% non-homeowner or renter	24.08% non-homeowner or renter	3750
Maternal smoking pre-pregnancy	26.93% any	2514	6.82%	27.11% any	37.07% any	3806

Maternal depression	22.78% any	2107	21.91%	23.62% any	24.09% any	1349
Parental separation	22.82% any	2371	12.12%	23.53% any	27.09% any	2370
Sexual abuse	3.31% any	2325	13.83%	3.53% any	2.97% any	2118
Physical abuse	38.76% any	2348	12.97%	38.39% any	26.24% any	2142
Age at menarche	12.66 years (1.11)	2620	2.89%	12.66 years	12.56 years (1.25)	1496
BMI	20.21 kg/m^2 (3.58)	2120	21.42%	20.40 kg/m^2	19.90 kg/m^2 (3.60)	1259
SDQ internal	2.59 points (2.60)	2236	17.12%	2.63 points	2.86 points (2.73)	1698
SDQ external	3.68 points (2.91)	2237	17.09%	3.74 points	3.88 points (2.87)	1697
IQ	104.59 score (15.57)	2092	22.46%	103.50 score	102.89 score (16.47)	1584

Abbreviations: SD, standard deviation; CSE, certificate of secondary education; BMI, body mass index; SDQ, strengths and difficulties questionnaire; IQ, intelligence quotient.

Supplementary Table 3. Linear regression analysis of the association between menstrual symptoms and school absences and GCSE score and logistic regression analysis of the association between menstrual symptoms and achieving five A*-C GCSEs including Maths and English and persistent absence (10% or more) (N=2698).

Continuous Outcomes						
**Exposure**	**School absence in Year 11**			**GCSE points score**		
	% change	95% CI	P value	Beta	95% CI	P Value
**Heavy or prolonged bleeding**						
Crude	22.71	15.20, 30.71	<0.001	-16.97	-22.70, -11.24	<0.001
Adjusted	16.58	9.43, 24.20	<0.001	-5.65	-10.10, -1.20	0.013
**Menstrual pain**						
Crude	16.28	9.37, 23.62	<0.001	-3.42	-8.99, 2.14	0.228
Adjusted	12.83	6.14, 19.95	0.001	-3.14	-7.46, 1.17	0.153
**Binary Outcomes**						
**Exposure**	**Persistent absence in Year 11**			**Five A*-C GCSEs incl. Maths & English**		
	OR	95% CI	P value	OR	95% CI	P Value
**Heavy or prolonged bleeding**						
Crude	1.69	1.65, 1.73	<0.001	0.60	0.59, 0.62	<0.001
Adjusted	1.48	1.45, 1.52	<0.001	0.73	0.71, 0.75	<0.001
**Menstrual pain**						
Crude	1.51	1.47, 1.54	<0.001	0.91	0.89, 0.92	<0.001
Adjusted	1.42	1.39, 1.46	<0.001	0.84	0.81, 0.86	<0.001

Abbreviations: GCSE, general certificate of secondary education; OR, odds ratio; CI, confidence interval. Adjusted for ethnicity; maternal education, parental social class, financial difficulties, and home ownership during pregnancy; maternal smoking pre-pregnancy; parental separation, physical abuse, and sexual abuse before 11; maternal depression at 12.1; age at menarche; body mass index at 12.8; internalising and externalising problems at 9.6; and intelligence quotient at 8.

Supplementary Table 4. Linear regression analysis of the association between different categories of menstrual symptoms and GCSE score and school absences (N=2698).

		**School absence in Year 11**			**GCSE points score**		
		% increase	95% CI	P value	Beta	95% CI	P Value
**Heavy or prolonged bleeding and visiting a doctor**							
Crude analysis	No symptom (ref) (N=2353)	0	-	-	0	-	-
	Symptom, no doctor (N=967)	15.21	7.68, 23.27	<0.001	-14.08	-20.23, -7.93	<0.0001
	Symptom and doctor (N=246)	57.36	39.53, 77.47	<0.001	-28.29	-39.22, -17.35	<0.0001
Adjusted analysis	No symptom (ref) (N=2353)	0	-	-	0	-	-
	Symptom, no doctor (N=967)	10.93	3.70, 18.67	0.003	-5.45	-10.23, -0.68	0.0252
	Symptom and doctor (N=246)	42.61	26.41, 60.89	<0.001	-5.92	-14.56, 2.72	0.1791
**Menstrual pain and visiting a doctor**							
Crude analysis	No symptom (ref) (N=1625)	0	-	-	0	-	-

	Symptom, no doctor (N=1616)	10.65	3.88, 17.86	0.002	-0.54	-6.30, 5.23	0.8552
	Symptom and doctor (N=292)	55.64	38.70, 74.64	<0.001	-20.08	-30.6, -9.57	0.0002
Adjusted analysis	No symptom (ref) (N=1625)	0	-	-	0	-	-
	Symptom, no doctor (N=1616)	8.76	2.13, 15.83	0.009	-2.53	-6.99, 1.94	0.2667
	Symptom and doctor (N=292)	42.30	26.74, 59.77	<0.001	-6.77	-15.00, 1.46	0.1068
**Heavy and prolonged, separately and combined**							
Crude analysis	Neither (ref) (N=2013)	0	-	-	0	-	-
	Prolonged only (N=286)	-8.48	-18.37, 2.61	0.129	4.78	-5.59, 15.15	0.366
	Heavy only (N=882)	20.74	12.46, 29.62	<0.001	-17.24	-23.69, -10.8	<0.001
	Both (N=307)	23.22	10.49, 37.42	<0.001	-13.73	-23.63, -3.84	0.007
Adjusted analysis	Neither (ref) (N=2013)	0	-	-	0	-	-
	Prolonged only (N=286)	-7.54	-17.39, 3.48	0.172	5.15	-2.84, 13.15	0.206
	Heavy only (N=882)	14.69	6.84, 23.13	<0.001	-4.82	-9.81, 0.16	0.058
	Both (N=307)	17.71	5.65, 31.14	0.003	-5.55	-13.19, 2.10	0.155
**Heavy and pain, separately and combined**							
Crude analysis	Neither (ref) (N=1280)	0	-	-	0	-	-
	Pain only (N=952)	10.44	2.31, 19.21	0.011	1.18	-5.76, 8.12	0.739
	Heavy only (N=276)	19.40	6.28, 34.13	0.003	-19.78	-30.35, -9.21	<0.001
	Both (N=920)	30.86	21.22, 41.26	<0.001	-15.46	-22.4, -8.51	<0.001
Adjusted analysis	Neither (ref) (N=1280)	0	-	-	0	-	-
	Pain only (N=952)	8.84	0.88, 17.42	0.029	-2.88	-8.24, 2.48	0.293
	Heavy only (N=276)	14.21	1.78, 28.14	0.024	-8.18	-16.34, -0.01	0.050
	Both (N=920)	23.13	14.02, 32.96	<0.001	-6.53	-11.94, -1.11	0.018

Abbreviations: GCSE, general certificate of secondary education; CI, confidence interval. Adjusted for ethnicity; maternal education, parental social class, financial difficulties, and home ownership during pregnancy; maternal smoking pre-pregnancy; parental separation, physical abuse, and sexual abuse before 11; maternal depression at 12.1; age at menarche; body mass index at 12.8; internalising and externalising problems at 9.6; and intelligence quotient at 8.

The following results are from analyses conducted in the complete case samples only.

Supplementary Table 5. Distribution of sociodemographic factors, adverse childhood experiences, and child factors according to adolescent menstrual symptoms in complete case sample only.

	Heavy or Prolonged Bleeding		Menstrual Pain	
	**Yes** ≤ 972 (36.03%)	**No** ≤ 1726 (63.97%)	**Yes** ≤ 1496 (55.45%)	**No** ≤ 1202 (44.55%)
**SOCIODEMOGRAPHIC FACTORS**				
**Ethnicity**				
White	830 (96.51)	1528 (96.16)	1301 (96.44)	1057 (96.09)
Non-white	30 (3.49)	61 (3.84)	48 (3.56)	43 (3.91)
**Maternal education**				
CSE/Vocational	195 (23.75)	306 (19.70)	276 (21.15)	225 (21.05)
O level	327 (39.83)	579 (37.28)	486 (37.24)	420 (39.29)
A level	214 (26.07)	407 (26.21)	354 (27.13)	267 (24.98)
Degree	85 (10.35)	261 (16.81)	189 (14.48)	157 (14.69)

Parental social class				
Manual	423 (50.78)	681 (44.31)	609 (46.56)	495 (46.61)
Non-manual	410 (49.22)	856 (55.69)	699 (53.44)	567 (53.39)
**Financial difficulties**				
Any	556 (64.95)	910 (58.15)	826 (61.60)	640 (59.26)
None	300 (35.05)	655 (41.85)	515 (38.40)	440 (40.74)
**Home ownership**				
Renter or non-homeowner	144 (16.36)	192 (12.00)	194 (14.10)	142 (12.86)
Owner or private renter	736 (83.64)	1408 (88.00)	1182 (85.90)	962 (87.14)
**Maternal smoking pre-pregnancy**				
Yes	277 (30.88)	400 (24.74)	409 (29.34)	268 (23.93)
No	620 (69.12)	1217 (75.26)	985 (70.66)	852 (76.07)
**ADVERSE CHILDHOOD EXPERIENCES**				
**Parental separation before age 11**				
Yes	212 (25.27)	329 (21.48)	300 (22.76)	241 (22.89)
No	627 (74.73)	1203 (78.52)	1018 (77.24)	812 (77.11)
**Physical abuse before age 11**				
Any	317 (38.33)	593 (38.99)	533 (40.69)	377 (36.32)
None	510 (61.67)	928 (61.01)	777 (59.31)	661 (63.68)
**Sexual abuse before age 11**				
Any	38 (4.67)	39 (2.58)	42 (3.28)	35 (3.36)
None	775 (95.33)	1473 (97.42)	1240 (96.72)	1008 (96.64)
**Maternal depression at 12.1 years**				
Yes	213 (29.42)	267 (19.31)	291 (25.15)	189 (19.89)
No	511 (70.58)	1116 (80.69)	866 (74.85)	761 (80.11)
**CHILD FACTORS**				
**Age at menarche**				
Mean (SE)	12.60 (1.12)	12.69 (1.10)	12.53 (1.08)	12.83 (1.31)
**BMI at 12.8 years**				
Mean (SE)	20.40 (3.54)	20.11 (3.59)	20.39 (3.16)	19.99 (3.64)
**Internalising SDQ at 9.6 years**				
Mean (SE)	2.74 (2.72)	2.51 (2.53)	2.76 (2.69)	2.38 (2.47)
**Externalising SDQ at 9.6 years**				
Mean (SE)	4.05 (3.08)	3.49 (2.79)	3.82 (2.91)	3.52 (2.91)
**IQ at 8 years**				
Mean (SE)	103.31 (15.86)	105.26 (15.38)	104.87 (15.36)	104.23 (15.83)
**Past year oral contraception at exposure timepoint**				
Yes	239 (24.95)	168 (9.85)	290 (19.58)	117 (9.89)
No	719 (75.05)	1538 (90.15)	1191 (80.42)	1066 (90.11)

Abbreviations: SE, standard error; CSE, certificate of secondary education; BMI, body mass index; SDQ, strengths and difficulties questionnaire; IQ, intelligence quotient.

Supplementary Table 6. Linear regression analysis of the association between menstrual symptoms and school absences and GCSE score and logistic regression analysis of the association between menstrual symptoms and achieving five A*-C GCSEs including Maths and English and persistent absence (10% or more) in the complete case sample only (N=1274).

Continuous Outcomes						
**Exposure**	**School absence in Year 11**			**GCSE points score**		
	% change	95% CI	P value	Beta	95% CI	P Value
**Heavy or prolonged bleeding**						
Crude	14.43	4.63, 25.14	0.003	-13.17	-20.36, -5.98	<0.001
Adjusted	11.24	1.78, 21.57	0.019	-6.12	-11.60, -0.64	0.029
**Menstrual pain**						
Crude	15.40	6.12, 25.48	0.001	-2.79	-9.56, 3.97	0.418
Adjusted	11.06	2.11, 20.79	0.015	-2.47	-7.67, 2.72	0.350
**Binary Outcomes**						
**Exposure**	**Persistent absence in Year 11**			**Five A*-C GCSEs incl. Maths & English**		
	OR	95% CI	P value	OR	95% CI	P Value
**Heavy or prolonged bleeding**						
Crude	1.41	1.05, 1.90	0.023	0.67	0.51, 0.88	0.004
Adjusted	1.27	0.93, 1.73	0.129	0.81	0.57, 1.14	0.218
**Menstrual pain**						
Crude	1.48	1.10, 1.99	0.009	0.94	0.72, 1.23	0.667
Adjusted	1.40	1.03, 1.90	0.032	0.92	0.66, 1.29	0.639

Abbreviations: GCSE, general certificate of secondary education; OR, odds ratio; CI, confidence interval. Adjusted for ethnicity; maternal education, parental social class, financial difficulties, and home ownership during pregnancy; maternal smoking pre-pregnancy; parental separation, physical abuse, and sexual abuse before 11; maternal depression at 12.1; age at menarche; body mass index at 12.8; internalising and externalising problems at 9.6; and intelligence quotient at 8.

Supplementary Table 7. Linear regression analysis of the associations between different categories of menstrual symptoms and school absences and GCSE score in the complete case sample only (N=1274).

		School absence in Year 11			GCSE points score		
		% increase	95% CI	P value	Beta	95% CI	P Value
**Heavy doctor**							
Crude analysis	No symptom (ref)	0	-	-	0	-	-
	Symptom, no doctor	8.03	-1.69, 18.71	0.108	-10.82	-18.42, -3.22	0.005
	Symptom and doctor	52.99	25.72, 86.17	<0.001	-24.99	-40.81, -9.17	0.002
Adjusted analysis	No symptom (ref)	0	-	-	0	-	-
	Symptom, no doctor	5.92	-3.54, 16.31	0.228	-5.84	-11.63, -0.05	0.048
	Symptom and doctor	42.22	17.03, 72.83	<0.001	-6.34	-18.41, 5.72	0.302
**Pain doctor**							
Crude analysis	No symptom (ref)	0	-	-	0	-	-
	Symptom, no doctor	11.38	2.25, 21.32	0.014	-0.45	-7.38, 6.47	0.898
	Symptom and doctor	55.47	29.64, 86.44	<0.001	-22.48	-37.18, -7.77	0.003
Adjusted analysis	No symptom (ref)	0	-	-	0	-	-
	Symptom, no doctor	7.97	-0.89, 17.62	0.079	-0.97	-6.27, 4.33	0.719
	Symptom and doctor	43.28	19.38, 71.95	0.001	-16.05	-27.34, -4.77	0.005
**Heavy and prolonged, separately and combined**							
Crude analysis	Neither (ref)	0	-	-	0	-	-
	Prolonged only	-9.63	-22.36, 5.18	0.191	1.77	-10.43, 13.96	0.776
	Heavy only	12.58	1.87, 24.43	0.020	-14.41	-22.45, -6.37	0.001
	Both	14.28	-2.95, 34.57	0.109	-8.04	-21.17, 5.10	0.230
Adjusted analysis	Neither (ref)	0	-	-	0	-	-
	Prolonged only	-8.82	-21.54, 5.96	0.228	5.40	-3.87, 14.67	0.253
	Heavy only	8.79	-1.49, 20.14	0.096	-6.38	-12.50, -0.26	0.041
	Both	13.79	-3.21, 33.78	0.117	-2.24	-12.22, 7.74	0.660
**Heavy and pain, separately and combined**							
Crude analysis	Neither (ref)	0	-	-	0	-	-
	Pain only	10.99	0.32, 22.79	0.043	3.59	-4.55, 11.72	0.388
	Heavy only	6.80	-9.56, 26.11	0.438	-5.92	-19.30, 7.46	0.385
	Both	24.44	11.63, 38.72	<0.001	-13.28	-22.02, -4.53	0.003
Adjusted analysis	Neither (ref)	0	-	-	0	-	-
	Pain only	7.10	-3.20, 18.49	0.184	0.74	-5.51, 6.98	0.817
	Heavy only	4.38	-11.42, 23.00	0.608	-1.44	-11.58, 8.70	0.781
	Both	18.32	6.14, 31.90	0.002	-7.16	-13.87, -0.46	0.036

Abbreviations: GCSE, general certificate of secondary education; CI, confidence interval. Adjusted for ethnicity; maternal education, parental social class, financial difficulties, and home ownership during pregnancy; maternal smoking pre-pregnancy; parental separation, physical abuse, and sexual abuse before 11; maternal depression at 12.1; age at menarche; body mass index at 12.8; internalising and externalising problems at 9.6; and intelligence quotient at 8.

Supplementary Table 8. Variables used to derive adverse childhood experiences (ACEs), including parental separation, physical abuse, and sexual abuse experienced up to age 11.

Variable ID	ACE Construct	Variable Description	Reported	Retrospective	Age reported	Start time period (years)	End time period (years)
f228	Parental separation	Divorce >CH born	Parent	No	8m	0	0.67
f237	Parental separation	Separation from PTNR >CH born	Parent	No	8m	0	0.67
g308	Parental separation	Mum divorced >CH8MTHs	Parent	No	1yrs9m	0.67	2
g317	Parental separation	Mum and partner separated >CH8MTHs	Parent	No	1yrs9m	0.67	2
h218	Parental separation	Whether mum got divorced since study child was 18 months old and effect this had	Parent	No	2yrs9m	1.5	3
h227	Parental separation	Whether mum and partner separated since study child was 18 months old and effect this had	Parent	No	2yrs9m	1.5	3
j308	Parental separation	MUM Divorced> CH 30 MTHs	Parent	No	3yrs11m	2.5	4
j317	Parental separation	MUM & PTR Separated> CH 30 MTHs	Parent	No	3yrs11m	2.5	4
k4008	Parental separation	Mother was divorced in past year	Parent	No	5yrs1m	4	5
k4017	Parental separation	Mother and partner separated in past year	Parent	No	5yrs1m	4	5
l4008	Parental separation	Respondent was divorced since study child's 5th birthday	Parent	No	6yrs1m	5	6
l4017	Parental separation	Respondent separated from partner since study child's 5th birthday	Parent	No	6yrs1m	5	6
p2008	Parental separation	Mother was divorced since the study child's 6th birthday	Parent	Yes	9yrs2m	6	7
p2017	Parental separation	Mother and husband/partner separated since the study child's 6th birthday	Parent	Yes	9yrs2m	6	7
r5008	Parental separation	Respondent has been divorced since child's 9th birthday	Parent	Yes	11yrs2m	9	10
r5017	Parental separation	Respondent separated from husband/partner since the study child's 9th birthday	Parent	Yes	11yrs2m	9	10
f246	Physical abuse	PTNR physically cruel to CHDR >CH born	Parent	No	8m	0	0.67
f247	Physical abuse	MUM physically cruel to CHDR >CH born	Parent	No	8m	0	0.67
g326	Physical abuse	Partner physically cruel to children >CH8MTHs	Parent	No	1yrs9m	0.67	2
g327	Physical abuse	Mum physically cruel to children >CH8MTHs	Parent	No	1yrs9m	0.67	2
h236	Physical abuse	Whether partner was physically cruel to children since study child was 18 months old and effect this had	Parent	No	2yrs9m	1.5	3
h237	Physical abuse	Whether mum was physically cruel to children since study child was 18 months old and effect this had	Parent	No	2yrs9m	1.5	3
j326	Physical abuse	PTR PHYS Cruel to CDRN> CH 30 MTHs	Parent	No	3yrs11m	2.5	4
j327	Physical abuse	MUM PHYS Cruel to CDRN> CH 30 MTHs	Parent	No	3yrs11m	2.5	4
k4026	Physical abuse	Mothers partner was physically cruel to children in past year	Parent	No	5yrs1m	4	5
k4027	Physical abuse	Mother was physically cruel to children in past year	Parent	No	5yrs1m	4	5
l4026	Physical abuse	Respondent's partner physically cruel to respondent's children since study child's 5th birthday	Parent	No	6yrs1m	5	6
l4027	Physical abuse	Respondent physically cruel to own children since study child's 5th birthday	Parent	No	6yrs1m	5	6
p2026	Physical abuse	Mother's husband/partner was physically cruel to her children since the study child's 6th birthday	Parent	Yes	9yrs2m	6	7
p2027	Physical abuse	Mother was physically cruel to her children since the study child's 6th birthday	Parent	Yes	9yrs2m	6	7
r5026	Physical abuse	Respondent's husband/partner was physically cruel to their children since study child's 9th birthday	Parent	Yes	11yrs2m	9	10

r5027	Physical abuse	Respondent was physically cruel to their children since the study child's 9th birthday	Parent	Yes	11yrs2m	9	10
ypb8002	Physical abuse	Frequency adult in family pushed, grabbed or shoved respondent before age of 11	Child	Yes	22yrs	0	11
ypb8003	Physical abuse	Frequency adult in family smacked respondent for discipline before age of 11	Child	Yes	22yrs	0	11
ypb8007	Physical abuse	Frequency adult in family hit respondent so hard it left bruises or marks before age of 11	Child	Yes	22yrs	0	11
kf455a	Sexual abuse	Child sexually abused > 18 months, Y/N	Parent	No	30m	1.5	2.5
kj465	Sexual abuse	CH was Sexually Abused Past 12 MTHs	Parent	No	42m	2.5	3.5
kl475	Sexual abuse	Child was sexually abused since age 3	Parent	No	57m	3	5
kn4005	Sexual abuse	Child sexually abused in past 15 months	Parent	No	69m	4.5	6
kq365	Sexual abuse	Child was sexually abused since his/her 5th birthday	Parent	No	81m	5	7
kt5005	Sexual abuse	Since 7th birthday child has been sexually abused	Parent	No	105m	7	9
ypb8030	Sexual abuse	Respondent was touched in a sexual way by adult or older child, or was forced to touch adult or older child in a sexual way, before age of 11	Child	Yes	22yrs	0	11
ypb8040	Sexual abuse	Adult or older child forced, or attempted to force, respondent into any sexual activity by threatening or holding respondent down or hurting respondent in some way, before age of 11	Child	Yes	22yrs	0	11

Information provided by Houtepen et al. (2018).[2] Binary ACE constructs were derived for parental separation, physical abuse, and sexual abuse for participants with data available on at least 50% of the relevant variables. Participants were categorised as experiencing 'any' parental separation, physical abuse, or sexual abuse if they have ever reported experiencing that ACE construct across any of the relevant variables between birth and age 11 years. Participants were categorised as experiencing 'no' parental separation, physical abuse, or sexual abuse if they have never reported experiencing that ACE construct across any of the relevant variables between birth and age 11 years.

Supplementary Table 9. **Linear regression analysis of the association between menstrual symptoms and school absences and GCSE score and logistic regression analysis of the association between menstrual symptoms and achieving five A*-C GCSEs including Maths and English and persistent absence (10% or more) adjusting for Key Stage 1 attainment (N=1120).**

Continuous Outcomes						
**Exposure**	**School absence in Year 11**			**GCSE points score**		
	% change	95% CI	P value	Beta	95% CI	P Value
**Heavy or prolonged bleeding**						
Adjusted for all confounders	12.19	2.05, 23.33	0.017	-5.73	-11.50, 0.04	0.052
Additionally adjusted for KS1 attainment	11.77	1.66, 22.89	0.021	-3.63	-9.02, 1.76	0.187
**Menstrual pain**						
Adjusted for all confounders	12.23	2.67, 22.68	0.011	-2.22	-7.68, 3.22	0.423
Additionally adjusted for KS1 attainment	12.26	2.70, 22.71	0.011	-2.37	-7.44, 2.69	0.358
**Binary Outcomes**						
**Exposure**	**Persistent absence in Year 11**			**Five A*-C GCSEs incl. Maths & English**		
	OR	95% CI	P value	OR	95% CI	P Value
**Heavy or prolonged bleeding**						
Adjusted for all confounders	1.21	0.87, 1.69	0.261	0.80	0.55, 1.14	0.215
Additionally adjusted for KS1 attainment	1.21	0.86, 1.69	0.271	0.88	0.60, 1.29	0.503
**Menstrual pain**						
Adjusted for all confounders	1.44	1.03, 2.01	0.032	0.85	0.60, 1.22	0.382
Additionally adjusted for KS1 attainment	1.44	1.03, 2.01	0.031	0.86	0.59, 1.24	0.415

Abbreviations: GCSE, general certificate of secondary education; KS1, Key Stage 1; OR, odds ratio; CI, confidence interval. Confounders include ethnicity; maternal education, parental social class, financial difficulties, and home ownership during pregnancy; maternal smoking pre-pregnancy; parental separation, physical abuse, and sexual abuse before 11; maternal depression at 12.1; age at menarche; body mass index at 12.8; internalising and externalising problems at 9.6; and intelligence quotient at 8.

Supplementary Table 10. Linear regression analysis of the association between menstrual symptoms and authorised school absences and logistic regression analysis of the association between menstrual symptoms and persistent authorised absence (10% or more) in the complete case sample only (N=1274).

Continuous Outcomes						
**Exposure**	**School absence (authorised and unauthorised) in Year 11**			**Authorised school absence in Year 11**		
	% change	95% CI	P value	% change	95% CI	P value
**Heavy or prolonged bleeding**						
Unadjusted	14.43	4.63, 25.14	0.003	14.38	4.71, 24.93	0.003
Adjusted	11.24	1.78, 21.57	0.019	11.25	1.89, 21.47	0.017
**Menstrual pain**						
Unadjusted	15.40	6.12, 25.48	0.001	13.54	4.53, 23.33	0.003
Adjusted	11.06	2.11, 20.79	0.015	9.23	0.52, 18.71	0.037
**Binary Outcomes**						
**Exposure**	**Persistent absence (authorised and unauthorised) in Year 11**			**Persistent authorised absence in Year 11**		
	OR	95% CI	P value	OR	95% CI	P Value
**Heavy or prolonged bleeding**						
Unadjusted	1.41	1.05, 1.90	0.023	1.32	0.96, 1.81	0.085
Adjusted	1.27	0.93, 1.73	0.129	1.19	0.86, 1.65	0.293
**Menstrual pain**						
Unadjusted	1.48	1.10, 1.99	0.009	1.35	0.99, 1.83	0.060
Adjusted	1.40	1.03, 1.90	0.032	1.27	0.92, 1.74	0.151

Abbreviations: OR, odds ratio; CI, confidence interval. Confounders include ethnicity; maternal education, parental social class, financial difficulties, and home ownership during pregnancy; maternal smoking pre-pregnancy; parental separation, physical abuse, and sexual abuse before 11; maternal depression at 12.1; age at menarche; body mass index at 12.8; internalising and externalising problems at 9.6; and intelligence quotient at 8.

Mathematical properties of odds ratios (ORs)

As outcomes are more common, ORs and risk ratios (RRs) diverge and many therefore argue that RRs should be used as they appear more intuitive. However, the properties of RRs mean they are not as intuitive as many believe and can lead to nonsensical conclusions and underestimations of the effect. Cook (2002) provides a comprehensive overview of these issues[1]. RRs are 'capped' by baseline prevalence, which can underestimate the effect. Cook provides an example comparing the effect of treatment in two populations with differing baseline risk of the disease. The high-risk population includes a control group with a risk of 60% and treatment group with a risk of 92.3%, resulting in a RR of 1.54 (OR of 8.0), and the low-risk population includes a control group with a risk of 4.5% and treatment group with a risk of 21.7%, resulting in a RR of 4.78 (OR of 5.83). Using the RRs, it is possible to draw conclusions that the effect of the treatment is substantially more damaging in the low risk group when it is actually plausible that the effect of treatment is similar between the groups, if not greater in the high-risk group. This is clear from the ORs and from a comparison of the absolute risk differences (32.3% in the high-risk group compared to 17.2% in the low-risk group). Whilst this is an extreme example, it clearly demonstrates how RRs can lead to underestimation of effects as the outcomes become more common. Similarly, it is argued that, with more common outcomes, it is less tenable to assume homogeneity in baseline risk amongst a population and, although both RRs and ORs are providing an average effect across this population, the RRs are more likely to result in nonsensical conclusions about individuals (or smaller subgroups within a population). For example, in this study, the crude RR for the association between heavy or prolonged bleeding and persistent absence would be 1.5. It is possible that within a population there are individuals (or groups) with a high baseline risk of persistent absence for a range of reasons. When this baseline risk exceeds 67%, the RRs will imply that their risk of persistent absence if exposed is 105% (or greater). ORs do not have this issue and, as nonsensical conclusions from RRs become more likely with higher baseline prevalence, it can be argued that they are preferred to RRs when outcomes are common. RRs also do not apply the rate of success or failure symmetrically, which ORs do, which can therefore result in different conclusions depending on whether the presence or absence of the outcome is being estimated. Therefore, whilst ORs and RRs diverge with more common outcomes, it is not always true that RRs should be the default as they can lead to underestimation and nonsensical conclusions.

STROBE Checklist

STROBE Statement—checklist of items that should be included in reports of observational studies

	**Item No**	**Recommendation**	**Included on page:**
**Title and abstract**	1	(*a*) Indicate the study's design with a commonly used term in the title or the abstract	1
		(*b*) Provide in the abstract an informative and balanced summary of what was done and what was found	2
**Introduction**			
Background/rationale	2	Explain the scientific background and rationale for the investigation being reported	3,4

Objectives	3	State specific objectives, including any pre-specified hypotheses	4
**Methods**			
Study design	4	Present key elements of study design early in the paper	2, 10-12
Setting	5	Describe the setting, locations, and relevant dates, including periods of recruitment, exposure, follow-up, and data collection	10-12
Participants	6	(*a*) *Cohort study*—Give the eligibility criteria, and the sources and methods of selection of participants. Describe methods of follow-up	10, 11
		Case-control study—Give the eligibility criteria, and the sources and methods of case ascertainment and control selection. Give the rationale for the choice of cases and controls	-
		Cross-sectional study—Give the eligibility criteria, and the sources and methods of selection of participants	-
		(*b*) *Cohort study*—For matched studies, give matching criteria and number of exposed and unexposed	-
		Case-control study—For matched studies, give matching criteria and the number of controls per case	-
Variables	7	Clearly define all outcomes, exposures, predictors, potential confounders, and effect modifiers. Give diagnostic criteria, if applicable	10-13
Data sources/ measurement	8*	For each variable of interest, give sources of data and details of methods of assessment (measurement). Describe comparability of assessment methods if there is more than one group	10-13
Bias	9	Describe any efforts to address potential sources of bias	14-18
Study size	10	Explain how the study size was arrived at	10, Figure 1
Quantitative variables	11	Explain how quantitative variables were handled in the analyses. If applicable, describe which groupings were chosen and why	11-14
Statistical methods	12	(*a*) Describe all statistical methods, including those used to control for confounding	14-18, Supplementary Figure 1
		(*b*) Describe any methods used to examine subgroups and interactions	-
		(*c*) Explain how missing data were addressed	15

		(*d*) *Cohort study*—If applicable, explain how loss to follow-up was addressed	15
		Case-control study—If applicable, explain how matching of cases and controls was addressed	-
		Cross-sectional study—If applicable, describe analytical methods taking account of sampling strategy	-
		(*e*) Describe any sensitivity analyses	15-18
Continued on next page			
**Results**			
Participants	13*	(a) Report numbers of individuals at each stage of study—eg numbers potentially eligible, examined for eligibility, confirmed eligible, included in the study, completing follow-up, and analysed	Figure 1
		(b) Give reasons for non-participation at each stage	Figure 1
		(c) Consider use of a flow diagram	Figure 1
Descriptive data	14*	(a) Give characteristics of study participants (eg demographic, clinical, social) and information on exposures and potential confounders	5, Table 1, Supplementary Table 2
		(b) Indicate number of participants with missing data for each variable of interest	Table 1, Supplementary Table 2, Supplementary Table 3
		(c) *Cohort study*—Summarise follow-up time (eg, average and total amount)	5
Outcome data	15*	*Cohort study*—Report numbers of outcome events or summary measures over time	Table 1
		Case-control study—Report numbers in each exposure category, or summary measures of exposure	-
		Cross-sectional study—Report numbers of outcome events or summary measures	-
Main results	16	(*a*) Give unadjusted estimates and, if applicable, confounder-adjusted estimates and their precision (eg, 95% confidence interval). Make clear which confounders were adjusted for and why they were included	5-6, Figure 2, Table 2, (Supplement Tables 4,5,6-10, page 1-2, Supplementary Figure 2)
		(*b*) Report category boundaries when continuous variables were categorized	-

		(*c*) If relevant, consider translating estimates of relative risk into absolute risk for a meaningful time period	-
Other analyses	17	Report other analyses done—eg analyses of subgroups and interactions, and sensitivity analyses	6 (supplement page 1-2)
**Discussion**			
Key results	18	Summarise key results with reference to study objectives	7
Limitations	19	Discuss limitations of the study, taking into account sources of potential bias or imprecision. Discuss both direction and magnitude of any potential bias	7-8
Interpretation	20	Give a cautious overall interpretation of results considering objectives, limitations, multiplicity of analyses, results from similar studies, and other relevant evidence	7-9
Generalisability	21	Discuss the generalisability (external validity) of the study results	7,9
**Other information**			
Funding	22	Give the source of funding and the role of the funders for the present study and, if applicable, for the original study on which the present article is based	19

Give information separately for cases and controls in case-control studies and, if applicable, for exposed and unexposed groups in cohort and cross-sectional studies.

Note: An Explanation and Elaboration article discusses each checklist item and gives methodological background and published examples of transparent reporting. The STROBE checklist is best used in conjunction with this article (freely available on the Web sites of PLoS Medicine at http://www.plosmedicine.org/, Annals of Internal Medicine at http://www.annals.org/, and Epidemiology at http://www.epidem.com/). Information on the STROBE Initiative is available at www.strobe-statement.org.

Supplementary References

1. Cook TD. Advanced statistics: Up with odds ratios! A case for odds ratios when outcomes are common. Academic Emergency Medicine. 2002. p. 1430–4.
2. Houtepen LC, Heron J, Suderman MJ, Tilling K, Howe LD. Adverse childhood experiences in the children of the avon longitudinal study of parents and children (ALSPAC). Wellcome Open Res. 2018;3.
